# Time Pressure and In-group Favoritism in a Minimal Group Paradigm

**DOI:** 10.3389/fpsyg.2020.603117

**Published:** 2020-11-12

**Authors:** Kaede Maeda, Hirofumi Hashimoto

**Affiliations:** ^1^Graduate School of Letters, Yasuda Women’s University, Hiroshima, Japan; ^2^Department of Social-Psychology, Yasuda Women’s University, Hiroshima, Japan

**Keywords:** decision time, group heuristic model, in-group favoritism, prisoner’s dilemma game (PDG), minimal group paradigm

## Abstract

Based on the group heuristic model and the model of intuitive cooperation, we hypothesized that in-group favoritism would be conspicuously shown through an intuitive process. To test this hypothesis, we utilized a minimal group paradigm, which is traditionally used in social psychological studies, and manipulated decision time in a one-shot prisoner’s dilemma game to compare the cooperative contribution level toward in-group and out-group members under three conditions: intuitive, empathic deliberation, and rational deliberation. Our findings confirmed that in-group favoritism was clearly shown in the intuitive condition only, suggesting that the intuitive cooperation model may only be valid in the context of social exchange with in-group members. Additional analysis also showed that in-group favoritism disappeared for participants who had been forced into empathic or rational deliberation for decision making. The theoretical implications of the findings are discussed.

## Introduction

Is human cooperation intuitive or does it requires deliberation? Recently, researchers, who take the position that intuition facilitates cooperative behavior, have shown that the shorter the time required for decision making, the more people show cooperative behavior in economic games (Rand et al., 2012, 2014). These findings appear to support the social heuristic hypothesis (Rand et al., 2014), which assumes that intuition drives people’s cooperation. However, the argument for the intuitive cooperation model is still being debated from various perspectives and has not been sufficiently concluded. Some research papers cast doubt on the robustness of the model (e.g., Tinghög et al., 2013; Kvarven et al., 2020) and others showed the conflicting findings (Capraro and Cococcioni, 2016). Furthermore, a research framework incorporating individual differences to understand the intuitive cooperation model has also been developed. Mischkowski and Glöckner (2016) focused on social value orientation and argued that intuitive cooperation was observed for prosocials, but not for proselfs. In addition, Yamagishi and colleagues argued that only individuals with prosocial orientation may react intuitively and cooperatively (Yamagishi et al., 2017; Andrighetto et al., 2020). These accumulating insights into intuitive cooperation, however, have not sufficiently examined the situational factors surrounding decision makers other than individual factors. In other words, previous studies have focused mainly on the decision makers themselves, that is, those who cooperate or not in experimental games, and little attention has been focused on the people they cooperate with, or what kind of social exchange is assumed by the decision makers. We speculate that whether decision makers cooperate in an economic game depends on who they interact with, and this validates arguments for the intuitive cooperation model. In the current study, therefore, we focused on shared group membership, which has not been the main focus of discussion of the intuitive cooperation model, except for a few studies (De Dreu et al., 2015; Everett et al., 2017).

Shared group membership can be a key factor affecting decisions in an economic game. A classic study of social psychology (e.g., Tajfel et al., 1971) has consistently shown that people tend to act more cooperatively with their own group members (in-group members) than with non-group members (out-group members). Standard understanding regarding in-group favoritism is based on social identity theory (e.g., Tajfel and Turner, 1979, 1986). However, the present study focuses on an alternative and evolutionary oriented explanation: the *group heuristic model* (Yamagishi et al., 1999, 2008b; Yamagishi and Kiyonari, 2000; Yamagishi, 2007). Yamagishi and colleagues proposed a group heuristic model arguing that people use a set of beliefs and decision rules *by default* among members belonging to the same community in a cooperative manner. As theoretical studies in mathematical biology explain the evolution of cooperation through indirect reciprocity (Nowak and Sigmund, 2005), especially through group structured (Masuda, 2012; Nax et al., 2015) or spatial structured indirect reciprocity (Li et al., 2019a,b), humans’ cooperation can be sustained within a group of individuals who behave in an indirectly reciprocal manner. Indirect reciprocity is considered a strategy of acting cooperatively toward people who have a reputation of being altruistic toward similar others. In terms of reputation within a group, the argument of the group heuristic model is applicable to the explanation of cooperation through indirect reciprocity; the group heuristic makes people behave to minimize the risk of exclusion from a reputation-based closed tight relationship. According to this group heuristic model, we predicted that the intuitive cooperation observed in previous studies is a reflection of a default decision strategy under the context of social exchange with in-group members. More specifically, combining the intuitive cooperation model and group heuristic model, we hypothesized that in-group favoritism would be conspicuously shown through an intuitive process (*hypothesis* 1). To examine *hypothesis* 1, we utilized a minimal group paradigm, which is traditionally used in social psychological studies, and experimentally manipulated decision time in one-shot anonymous interactions [i.e., the prisoners’ dilemma game (PDG)] with in-group and out-group members.

The secondary aim of the present study was to examine the potential effect of deliberation. According to most previous studies discussing the model of intuitive cooperation, it seems to be assumed that the deliberation process overrides intuitive cooperation, and the findings suggest that cooperative decisions occur more quickly than deliberatively (e.g., Rand et al., 2012). Granting that intuition favors cooperation, it does not necessarily mean that deliberation will always prevent cooperation. Specifically, it depends on whether the deliberation was forced empathically or rationally. Batson and colleagues (Batson and Moran, 1999; Batson and Ahmad, 2001) demonstrated that manipulation of empathy enhanced people’s cooperation level in the PDG, suggesting that empathic deliberation promotes cooperation. We therefore examined the effect of two types of deliberation: empathic deliberation and rational deliberation. When it is said that cooperation is prevented by deliberation, deliberation is thought to refer to a case of playing the PDG after rational consideration, that is maximizing own profit. The present study defines this type of deliberation in terms of *rational deliberation*. There is, of course, another type of deliberation in which the player thinks of the emotions of the other party in the PDG. This type of deliberation is defined as *empathic deliberation*. It can be expected that cooperative contribution would be difficult if rational deliberation was forced. On the other hand, empathic deliberation increases the level of cooperative contribution. More important here is the potential effect of these two types of deliberation on in-group favoritism. Based on the argument of “empathy’s narrow focus” (Bloom, 2017), the extent to which empathic deliberation can work may be bounded with in-group members. In other words, empathic deliberation is less likely to lead to cooperative behavior toward out-group members. We then hypothesized that in-group favoritism would be enhanced by the empathic deliberation process as well as the intuitive process (*hypothesis* 2a). Additionally, it was predicted that rational deliberation would lower the level of cooperation even among in-group members, and as a result, in-group favoritism would disappear as a result of lowering the overall cooperative contribution (*hypothesis* 2b). Analyzing the potential effects of these two kinds of deliberation is also the secondary purpose of the current study.

In summary, the current study hypothesized that in-group favoritism is intuitive, and therefore, in-group favoritism in a minimal group would be more salient with an intuitive process (*hypothesis* 1). We also assumed two potential effects of deliberation on in-group favoritism. Specifically, we hypothesized that empathic deliberation promotes in-group bias (*hypothesis* 2a) and that rational deliberation decreases overall cooperation level, and as a result, eliminates in-group bias (*hypothesis* 2b). To test these hypotheses, we utilized a minimal group paradigm and manipulated decision time in the PDG to compare contribution levels toward in-group and out-group members under three conditions, that is, intuitive process, empathic deliberation, and rational deliberation.

## Materials and Methods

### Participants

This study was conducted as part of a supplementary lecture on social psychology, and lecture attendants were invited to participate. A total of 102 Japanese female undergraduates (mean age = 19.851, SD = 0.713) voluntarily participated.

### Experimental Design

We used a 3 (condition: intuitive, empathic deliberation, and rational deliberation) × 2 (group membership: in-group and out-group) between-subjects factorial design.

### Minimal Group Paradigm and One-Shot Prisoners’ Dilemma Game

According to the minimal group paradigm, we arbitrarily divided participants into two groups, in this case according to a painting preference task. Nine pairs of paintings by renowned artists Paul Klee and Wassily Kandinsky were presented on a screen using PowerPoint slides for easy viewing, and participants were asked to answer which painting they preferred. Based on the results of this preference task, participants were divided to either the Klee or Kandinsky group and given a questionnaire in a closed envelope corresponding to their belonging group. After confirming that all participants received the questionnaire, the experimenter explained the general rules of the one-shot PDG: (1) Participants were paired to play the game. (2) Participants were allocated 500 JPY (about $5) from the experimenter and were asked how much money they would give to their counterpart/other party based on the provisions of 3–5. (3) Participants were told that the amount of money given to their counterparts would be doubled by the experimenter and offered to the counterparts as a reward. (4) The amount of money left in the hands of the participant, without being offered to the other party, would be the participant’s own reward, but would not be doubled. (5) Their counterpart made exactly the same decision. In this study, it was emphasized that 15% of the participants would be given the money determined by their actual decisions in this one-shot PDG.

### Manipulation of Decision Time and Types of Deliberation

Participants were randomly assigned to the intuitive (*n* = 33), empathic deliberation (*n* = 33), and rational deliberation (*n* = 36) conditions and played the one-shot PDG with a member of their own group (in-group condition) or with a member of the other group (out-group condition). After hearing the above explanation, participants were asked to note their decisions of how much money they would give to the other party. In the intuitive condition, participants were asked to make a decision within 5 s. Aligned to the procedures followed by Batson and colleagues (Batson and Moran, 1999; Batson and Ahmad, 2001), we attempted to induce participants’ empathy in the empathic deliberation condition. Specifically, before making their decisions, participants were asked to deliberate for 3 min on what they and their counterparts would feel (happy or sad) regarding the potential consequences of each player’s decision. Similarly, in the rational deliberation condition, before making a decision, participants were asked to deliberate for 3 min on whether they and the other party would lose or gain payoffs from the combination of possible outcomes in the one-shot PDG. Through these manipulations, we attempted to induce contexts for deliberation. After participants noted their decisions on the questionnaire, they all were asked how much they expected the other party would contribute. According to the group heuristic model, it is the expectation of the other party’s cooperation that can yield in-group favoritism (Yamagishi et al., 2008b). In the context of social exchange with in-group members, it is expected that the same community members will behave in a cooperative manner. To confirm this, we measured participants’ expectations of how the other party would behave in the one-shot PDG.

## Results

### The Average Contribution in One-Shot Prisoners’ Dilemma Game

**Figure 1:**
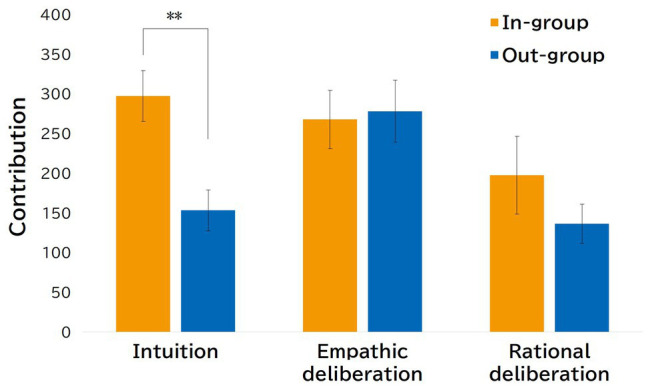
Average contribution in the PDG by three conditions (error bars are standard errors). ^**^p < 0.01 and ^*^p < 0.05.

Figure 1 summarizes the descriptive statistics of average contribution in the PDG for each condition by the group membership of counterparts. We conducted a 3 (condition: intuitive, empathic deliberation, and rational deliberation) × 2 (group membership: in-group and out-group) ANOVA of average contribution of PDG. The main effect of group membership [*F*(1, 96) = 5.301, *p* = 0.023, partial *η*^2^ = 0.052] and the main effect of condition [*F*(2, 96) = 4.803, *p* = 0.010, partial *η*^2^ = 0.091] were significant, although the interaction effect of group membership and condition was not significant [*F*(2, 96) = 2.460, *p* = 0.091, partial *η*^2^ = 0.049]. To clarify the main effect of condition, we performed an additional multiple comparison analysis and found significant differences in the average contribution of the PDG between the empathic deliberation condition and rational deliberation condition [*t*(96) = 3.091, *p* = 0.003]. We also performed an additional multiple comparison analysis for each condition to examine the extent of in-group favoritism and found significant differences in the average contribution of the PDG only in the intuition condition [*t*(96) = 2.922, *p* = 0.004]. No significant differences were observed in the empathic deliberation condition [*t*(96) = 0.213, *ns.*] and the rational deliberation condition [*t*(96) = 1.278, *ns.*]. These results support *hypothesis* 1 and *hypothesis* 2b, but did not support *hypothesis* 2a.

### Expectations

**Figure 2:**
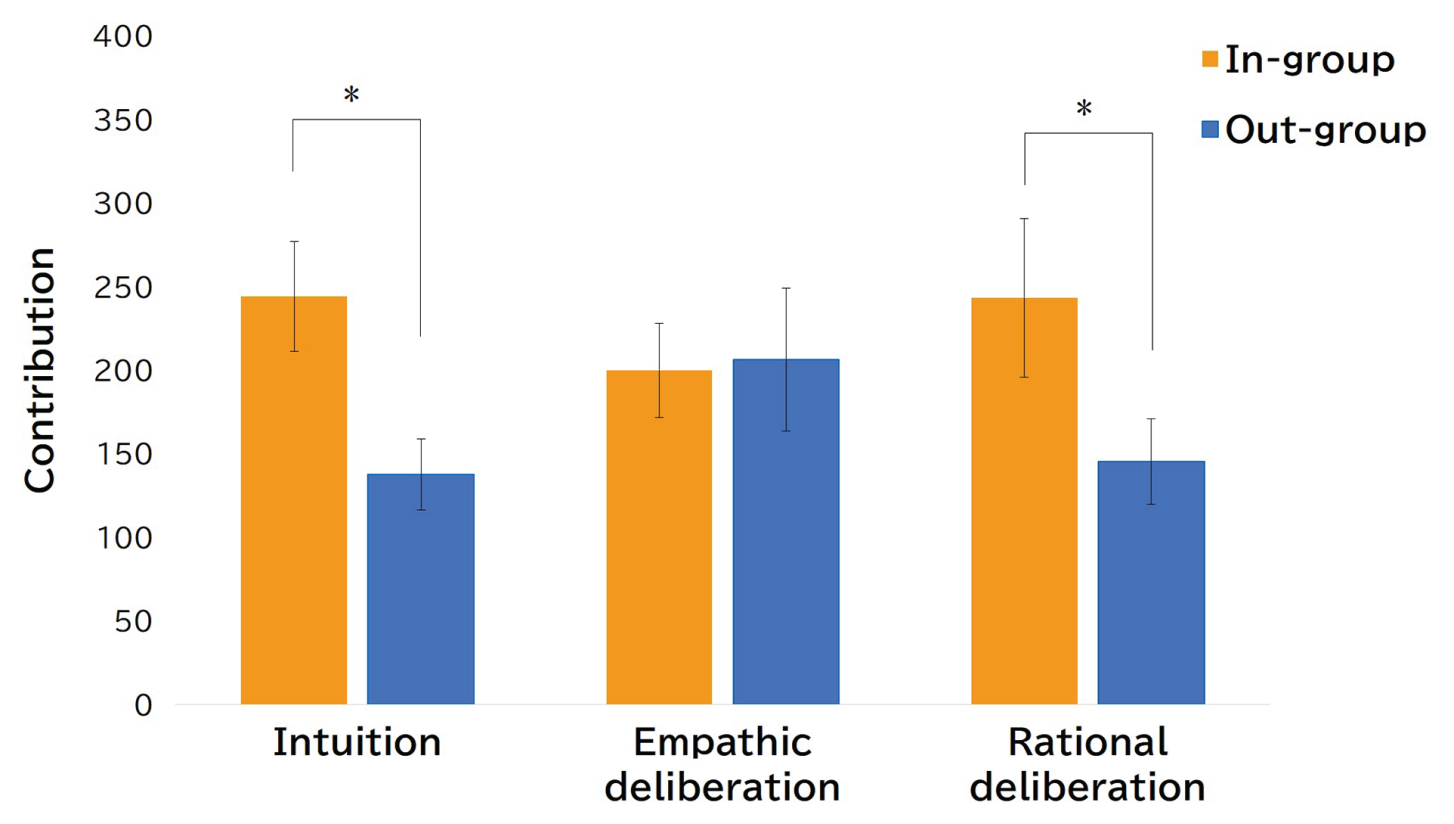
Average expected contribution in the PDG by three conditions (error bars are standard errors). ^**^p < 0.01 and ^*^p < 0.05.

Figure 2 demonstrates the descriptive statistics of the average expected contribution in the PDG for each condition by the group membership of other parties. The pattern in Figure 2 seemed to be almost identical to the pattern shown in Figure 1. We conducted the same ANOVA of average expected contribution in PDG and found that the main effect of group membership [*F*(1, 96) = 5.867, *p* = 0.017, partial *η*^2^ = 0.058] was significant. No other effects were significant. Additional multiple comparison analysis by each condition to examine the extent of in-group favoritism found that there were significant differences in the intuition condition [*t*(96) = 2.232, *p* = 0.028] and rational deliberation condition [*t*(96) = 2.115, *p* = 0.037]. No significant differences were observed in the empathic deliberation condition [*t*(96) = 0.131, *ns.*]. What should be noted here is the observed in-group bias of participants’ expectations in the rational deliberation condition. Participants who were assigned to this condition and played the PDG with in-group members expected other parties to behave cooperatively, while their own cooperative contribution was at a low level. These results suggest that rational deliberation regarding payoff gains and losses in the PDG eliminates in-group bias in the form of lowering cooperation toward in-group members. On the other hand, in the intuitive condition, both expectation and actual cooperative action showed in-group favoritism, which indirectly supports *hypothesis* 1, in which intuitive cooperation is understood as a reflection of default decision strategy in the context of social exchange with in-group members.

## General Discussion

In the current study, we focused on the association between peoples’ cooperative behavior and decision time in the context of social exchange with in-group or out-group members. Our findings provide evidence that in-group favoritism was clearly shown in intuitive conditions, which supports *hypothesis* 1. However, our results did not support *hypothesis* 2a, although they supported *hypothesis* 2b. Specifically, both types of deliberation cause the in-group bias to be absent, although empathic deliberation increased the overall rate of cooperation in the PDG, whereas rational deliberation decreased overall cooperation rates. Interestingly, the cooperation rates toward in-group members in the intuitive condition were equivalent to the cooperation rates in the empathic deliberation condition, whereas the cooperation rates toward out-group members in the intuitive condition were virtually identical to the cooperation rates in the rational deliberation condition. This pattern of results suggests that people tend to intuitively cooperate with in-group members, not out-group members, at the same level as empathically deliberated cooperation. A recent meta-analysis study suggests that emotion-inducing manipulations used in experimental procedures is an important key in considering the relationship between intuition and cooperation (Kvarven et al., 2020). In this sense, the results of the current study are suggestive in terms of showing that the cooperative contribution toward in-group members in the intuition condition indicates a pattern similar to the results observed in empathic deliberation conditions. Many recent studies have attempted to understand cognitive processes underpinning human sociality including cooperation (for review, Capraro, 2019). Our study findings, although narrowly focusing on cooperation and shared group membership, showed that individuals may be intuitive cooperators, but only in the context of cooperation toward in-group members. That is, our findings add new experimental evidence for intuitive cooperation.

Several issues need to be addressed. One issue is a more adequate way to examine the potential effect of emphatic deliberation. We set the emphatic deliberation based on the arguments regarding “empathy’s narrow focus.” However, we did not set the incentive structure in zero-sum game situations between in-group and out-group members. Batson et al.’s intriguing study (Batson et al., 1995) argued the immorality of empathy by setting up the situation that in-group favoritism creates the unfortunate situation of out-group members. In the current study, such a zero-sum situation was not set up. Therefore, it is worth examining whether empathic deliberation with in-group favoritism will disappear even in a zero-sum situation. Another issue concerns the limited sample size of the current study, and its weakness in terms of restricted gender, age, and culture. Specifically, our results may be limited to young Japanese female students. Rand et al. pointed out gender differences in altruistic behavior and suggested that intuition favors cooperation with women (Rand et al., 2016; but see also Rand, 2017). Therefore, there is no denying the possibility that the findings of the current study are based on the fact that the sample was limited to female university students. We should also note that we recruited only university students regardless of gender. Matsumoto et al. (2016) actually pointed out the possibility that people’s cooperation in economic games increases as age increases. Then, the effect of age on intuitive cooperation should also be examined more carefully. Additionally, potential cultural differences in intuitive cooperation toward in-group members is an interesting issue to examine. According to the group heuristic model, it is understandable that intuitive cooperation toward in-group members is a default strategy to minimize the risk of being excluded from one’s group or relationships (Yamagishi et al., 2008a). According to the social niche construction theory (Yamagishi and Hashimoto, 2016), East Asian societies are characterized as closed collectivistic societies where security within particular groups and relationships provide individuals with their required resources, and there are few to no alternative external sources that individuals can access for resources (e.g., Greif, 1994; Hashimoto and Yamagishi, 2016). Accordingly, it is expected that intuitive cooperation toward in-group members would be more prominent in East Asian societies, like Japan. Of course, this is just speculation at this point. Although the validation of explanation based on the group heuristic model for in-group favoritism has been shown to apply to non-Japanese people as well (e.g., Foddy et al., 2009; Platow et al., 2012), future research will need to include participants from different cultures and with different demographics. Finally, it is also important to note that the current study uses the PDG, which is played pairwise. It might be more appropriate to use the public goods game, which is played among n-person rather than the PDG, in that we are trying to understand the reciprocity within a group. While the differences of game types (i.e., PDG or public goods game) potentially affect the relationship between intuition, group membership, and people’s cooperative behavior, according to Rand et al. (2016) arguments, it would be possible to predict that the difference of game types will not have much effect because both are non-zero-sum games. However, whether the findings of the current study using PDG can be replicated using the public goods game is an important question to examine in future research.

Despite these limitations, the current study contributes to our understanding of humans’ cooperative behaviors. As stated in the Introduction section, the shared group membership, which has been attracting attention in the field of social psychology, has been incorporated into the discussion of the model of intuitive cooperation in recent years, and we believe that such an incorporation can give theoretical implications not only for social psychologists, but also for social scientists, for example, who are considering cooperative or moral behaviors from the viewpoints of social physics (e.g., Capraro and Perc, 2018) and/or evolutionally game-theoretically oriented social science (e.g., Gintis, 2009; Bowles and Gintis, 2011).

## Data Availability Statement

The raw data supporting the conclusions of this article will be made available by the authors, without undue reservation.

## Ethics Statement

The studies involving human participants were reviewed and approved by Yasuda Women’s University. The patients/participants provided their written informed consent to participate in this study.

## Author Contributions

KM and HH contributed to the study design, analyzed data, and wrote the whole part of manuscript. KM conducted data collection. Both the authors contributed to the article and approved the submitted version.

### Conflict of Interest

The authors declare that the research was conducted in the absence of any commercial or financial relationships that could be construed as a potential conflict of interest.

## References

[ref1] Andrighetto G. Capraro V. Guido A. Szekely A. (2020). Cooperation, response time, and social value orientation a meta-analysis. PsyArXiv [Preprint]. 10.31234/osf.io/cbakz

[ref2] Batson C. D. Ahmad N. (2001). Empathy-induced altruism in a prisoner’s dilemma II: what if the target of empathy has defected? Eur. Rev. Soc. Psychol. 31, 25–36. 10.1002/ejsp.26

[ref3] Batson C. D. Klein T. R. Highberger L. Shaw L. L. (1995). Immorality from empathy-induced altruism: when compassion and justice conflict. J. Pers. Soc. Psychol. 68, 1042–1054. 10.1037/0022-3514.68.6.1042

[ref4] Batson C. D. Moran T. (1999). Empathy-induced altruism in a prisoner’s dilemma. Eur. Rev. Soc. Psychol. 29, 909–924. 10.1002/(SICI)1099-0992(199911)29:7<909::AID-EJSP965>3.0.CO;2-L

[ref5] Bloom P. (2017). Against empathy: The case for rational compassion. New York, NY: Penguin Random House.

[ref6] Bowles S. Gintis H. (2011). A cooperative species: Human reciprocity and its evolution. Princeton, NJ: Princeton University Press.

[ref7] Capraro V. (2019). The dual-process approach to human sociality: A review. 10.1037/pspa0000375 38227465

[ref8] Capraro V. Cococcioni G. (2016). Rethinking spontaneous giving: extreme time pressure and ego-depletion favor self-regarding reactions. Sci. Rep. 6:27219. 10.1038/srep27219, PMID: 27251762 PMC4890119

[ref9] Capraro V. Perc M. (2018). Grand challenges in social physics: in pursuit of moral behavior. Front. Phys. 6:107. 10.3389/fphy.2018.00107

[ref10] De Dreu C. K. W. Dussel D. B. Ten Velden F. S. (2015). In intergroup conflict, self-sacrifice is stronger among pro-social individuals, and parochial altruism emerges especially among cognitively taxed individuals. Front. Psychol. 6:572. 10.3389/fpsyg.2015.00572, PMID: 25999888 PMC4422010

[ref11] Everett J. A. C. Ingbretsen Z. Cushman F. Cikara M. (2017). Deliberation erodes cooperative behavior—even towards competitive out-groups, even when using a control condition, and even when eliminating selection bias. J. Exp. Soc. Psychol. 73, 76–81. 10.1016/j.jesp.2017.06.014

[ref12] Foddy M. Platow M. J. Yamagishi T. (2009). Group-based trust in strangers: the role of stereotypes and expectations. Psychol. Sci. 20, 419–422. 10.1111/j.1467-9280.2009.02312.x, PMID: 19399956

[ref13] Gintis H. (2009). The bounds of reason: Game theory and the unification of the behavioral sciences. Princeton, NJ: Princeton University Press.

[ref14] Greif A. (1994). Cultural beliefs and the organization of society: a historical and theoretical reflection on collectivist and individualist societies. J. Polit. Econ. 102, 912–950. 10.1086/261959

[ref15] Hashimoto H. Yamagishi T. (2016). Duality of independence and interdependence: an adaptationist perspective. Asian J. Soc. Psychol. 19, 286–297. 10.1111/ajsp.12145

[ref16] Kvarven A. Strømland E. Wollbrant C. Andersson D. Johannesson M. Tinghög G. (2020). The intuitive cooperation hypothesis revisited: a meta-analytic examination of effect size and between-study heterogeneity. J. Econ. Sci. Assoc. 6, 26–42. 10.1007/s40881-020-00084-3

[ref17] Li X. Sun S. Xia C. (2019a). Reputation-based adaptive adjustment of link weight among individuals promotes the cooperation in spatial social dilemmas. Appl. Math. Comput. 361, 810–820. 10.1016/j.amc.2019.06.038

[ref18] Li X. Wang H. Xia C. Perc M. (2019b). Effects of reciprocal rewarding on the evolution of cooperation in voluntary social dilemmas. Front. Phys. 7:125. 10.3389/fphy.2019.00125, PMID: 12004134

[ref19] Masuda N. (2012). Ingroup favoritism and intergroup cooperation under indirect reciprocity based on group reputation. J. Theor. Biol. 311, 8–18. 10.1016/j.jtbi.2012.07.002, PMID: 22796271

[ref20] Matsumoto Y. Yamagishi T. Li Y. Kiyonari T. (2016). Prosocial behavior increases with age across five economic games. PLoS One 11:e0158671. 10.1371/journal.pone.0158671, PMID: 27414803 PMC4945042

[ref21] Mischkowski D. Glöckner A. (2016). Spontaneous cooperation for prosocials, but not for proselfs: social value orientation moderates spontaneous cooperation behavior. Sci. Rep. 6:21555. 10.1038/srep21555, PMID: 26876773 PMC4753511

[ref22] Nax H. H. Perc M. Szolnoki A. Helbing D. (2015). Stability of cooperation under image scoring in group interactions. Sci. Rep. 5:12145. 10.1038/srep12145, PMID: 26177466 PMC4502532

[ref23] Nowak M. A. Sigmund K. (2005). Evolution of indirect reciprocity. Nature 437, 1291–1298. 10.1038/nature04131, PMID: 16251955

[ref24] Platow M. J. Foddy M. Yamagishi T. Lim L. Chow A. (2012). Two experimental tests of trust in in-group strangers: the moderating role of common knowledge of group membership. Eur. J. Soc. Psychol. 42, 30–35. 10.1002/ejsp.852

[ref25] Rand D. G. (2017). Social dilemma cooperation (unlike dictator game giving) is intuitive for men as well as women. J. Exp. Soc. Psychol. 73, 164–168. 10.1016/j.jesp.2017.06.013 29686434 PMC5909828

[ref26] Rand D. G. Brescoll V. L. Everett J. A. C. Capraro V. Barcelo H. (2016). Social heuristics and social roles: intuition favors altruism for women but not for men. J. Exp. Psychol. Gen. 145, 389–396. 10.1037/xge0000154, PMID: 26913619

[ref27] Rand D. G. Greene J. D. Nowak M. A. (2012). Spontaneous giving and calculated greed. Nature 489, 427–430. 10.1038/nature11467 22996558

[ref28] Rand D. G. Peysakhovich A. Kraft-Todd G. T. Newman G. E. Wurzbacher O. Nowak M. A.. (2014). Social heuristics shape intuitive cooperation. Nat. Commun. 5:3677. 10.1038/ncomms4677, PMID: 24751464

[ref29] Tajfel H. Billig M. G. Bundy R. P. Flament C. (1971). Social categorization and intergroup behaviour. Eur. J. Soc. Psychol. 1, 149–178. 10.1002/ejsp.2420010202

[ref30] Tajfel H. Turner J. C. (1979). “An integrative theory of intergroup conflict” in The psychology of intergroup relations. eds. Austin W. G. Worchel S. (Nelson-Hall: Monterey), 33–47.

[ref31] Tajfel H. Turner J. C. (1986). “The social identity theory of intergroup behavior” in Psychology of intergroup behavior. eds. Worchel S. Austin W. G. (Chicago: Nelson Hall), 7–24.

[ref32] Tinghög G. Andersson D. Bonn C. Böttiger H. Josephson C. Lundgren G.. (2013). Intuition and cooperation reconsidered. Nature 498, 427–430. 10.1038/nature12194, PMID: 23739429

[ref33] Yamagishi T. (2007). “The social exchange heuristic: a psychological mechanism that makes a system of generalized exchange self-sustaining” in Cultural and ecological foundations of the mind. eds. Radford M. Ohnuma S. Yamagishi T. (Sapporo: Hokkaido University Press), 11–37.

[ref34] Yamagishi T. Hashimoto H. (2016). Social niche construction. Curr. Opin. Psychol. 8, 119–124. 10.1016/j.copsyc.2015.10.003 29506786

[ref35] Yamagishi T. Hashimoto H. Schug J. (2008a). Preferences versus strategies as explanations for culture-specific behavior. Psychol. Sci. 19, 579–584. 10.1111/j.1467-9280.2008.02126.x 18578848

[ref36] Yamagishi T. Jin N. Kiyonari T. (1999). Bounded generalized reciprocity. Adv. Group Process. 16, 161–197.

[ref37] Yamagishi T. Kiyonari T. (2000). The group as the container of generalized reciprocity. Soc. Psychol. Q. 63, 116–132. 10.2307/2695887

[ref38] Yamagishi T. Matsumoto Y. Kiyonari T. Takagishi H. Li Y. Kanai R.. (2017). Response time in economic games reflects different types of decision conflict for prosocial and proself individuals. Proc. Natl. Acad. Sci. U. S. A. 114, 6394–6399. 10.1073/pnas.1608877114, PMID: 28559334 PMC5474786

[ref39] Yamagishi T. Mifune N. Liu J. H. (2008b). Exchanges of group-based favours: Ingroup bias in the prisoner’s dilemma game with minimal groups in Japan and New Zealand. Asian J. Soc. Psychol. 11, 196–207. 10.1111/j.1467-839X.2008.00258.x

